# Outcomes of U-shaped internal fixation in the treatment of avulsion fracture of calcaneal tubercle

**DOI:** 10.1186/s12891-023-06542-3

**Published:** 2023-05-22

**Authors:** Weigang Lou, Min Liu, Ding Xu, Ming Li, Jianming Chen

**Affiliations:** 1 Department of Orthopedic Trauma Surgery, Ningbo NO.6 Hospital, Ningbo, China; 2 Department of Orthopedics, Third Affiliated Hospital of Wenzhou Medical University, Wenzhou, China

**Keywords:** Avulsion fracture of calcaneal tubercle, Efficacy, U-shaped internal fixation

## Abstract

**Background:**

The purpose of this study was to analyze the efficacy of U-shaped internal fixation for calcaneal tubercle fracture after nearly 3 years of case follow-up and data collection.

**Method:**

We retrospectively analyzed the collected data from 16 patients with avulsion fracture of calcaneal tubercle between December 2018 and February 2021 at our institute. All patients were required to conform to regular follow up postoperatively. X-ray film was applied to all cases. The American Orthopaedic Foot and Ankle Association (AOFAS) score, Cedell score and the visual analog scale (VAS) were used to evaluate functional results.

**Results:**

All patients achieved bone union. The preoperative AOFAS score was 26.34 ± 3.34, which was significantly different from 91.38 ± 6.15 half a year after operation (p = 0.003). The preoperative Cedell score was 31.05 ± 4.18 and the score half a year after operation was 92.17 ± 5.39(p = 0.011). The VAS score was 8.91 ± 1.51 before operation and decreased to 0.58 ± 1.31 half a year after operation (p = 0.014).

**Conclusions:**

In the treatments of calcaneal tubercle fracture, U-shaped internal fixation is a new attempt. Through the short-term follow-up study, we found that its therapeutic effect is excellent, which is a recommended treatment in clinic.

## Introduction

Calcaneal tubercle is an important part of heel, which is of great significance in maintaining foot arch and buffering weight [1, 2]. Avulsion tubercle fracture of calcaneus is common in the elderly and athletes. when the ankle is strongly dorsiflexed or under direct stress, the elderly often has avulsion fractures due to osteoporosis and the decline of mechanical properties, which lead to the calcaneus unable to bear the traction of Achilles tendon. This is different from the calcaneal tubercle fracture caused by longitudinal violence in young athletes [3, 4]. The incidence of avulsion fracture of calcaneal tubercle is not high, it only accounts for 1.3% ~ 2.7% of all calcaneal fractures [5]. Due to the traction of Achilles tendon, the fracture displacement of calcaneal tubercle is often large, which is easy to cause excessive local tension or soft tissue irritation, resulting in local flap necrosis. If the condition worsens, the calcaneus and achilles tendon may be exposed [6]. Therefore, early surgical intervention is helpful to reduce the incidence of soft tissue necrosis and restore the function of triceps and the ability of metatarsal foot as soon as possible. These are of great significance to the rehabilitation of patients [7]. However, the opinions of scholars on which internal fixation method should be used for calcaneal tubercle fracture are not unified. The main clinical methods are internal fixation with wire anchor, screw and calcaneal plate [8–11]. Due to the traction of Achilles tendon and the special anatomical structure of the surgical site, high requirements are put forward for the firmness of internal fixation. It is reported that many cases of calcaneal tubercle fracture have complications such as failure of internal fixation, re-displacement of fracture, bone resorption and skin necrosis [12, 13]. Need for secondary surgery due to various complications ranged from 20 to 36.3% in the literature [14, 15]. Carnero martín et al. found that the incidence of complications after treatment was about 30% when the displacement of fracture block was less than 2 cm. When the displacement was greater than or equal to 2 cm, the probability of complications will soar to 90%, especially those involving soft tissue [16]. We have proposed a new and safe U-shaped internal fixation method (180-degree annular internal fixation) for the treatment of calcaneal tubercle fractures [17]. To analyze the efficacy of this treatment method for calcaneal tubercle fracture after nearly 3 years of case follow-up and data collection, we hope to provide a new treatment option for clinicians.

## Patients and methods

The avulsion fracture of calcaneal tubercle was classified using the method described by Beavis et al. [18]. The inclusion criteria for the research were as follows: [1] acute, avulsion fracture of calcaneal tubercle (grade I and II) determined according to the classification described by Beavis et al.; [2] no more than 14 days of trauma; [3] signed informed consent. The exclusion criteria were as follows: [1] other types (grade III); [2] no signed informed consent; [3] previous surgery on the heel; [4] history of avulsion fracture of calcaneal tubercle or other heel trauma.

We retrospectively analyzed the collected data from 16 patients with avulsion fracture of calcaneal tubercle between December 2018 and February 2021 at Ningbo No.6 Hospital. All cases were performed by two surgeons (WGL and DX). The research protocol was approved by the Institutional Review Board of Ningbo No.6 Hospital. Written informed consent was obtained from all of the participants, and the research methods were carried out in accordance with approved guidelines.

### Surgical methods

**Fig. 1:**
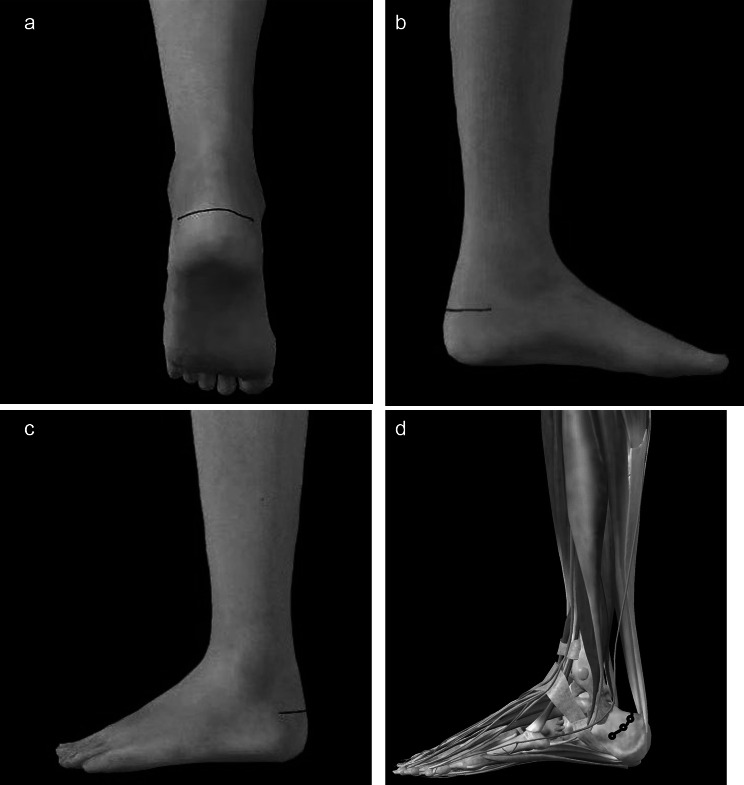
**a.b.c**: Schematic diagram of the surgical incision. A 4-5 cm U-shaped incision was made on the horizontal plane at the level of the calcaneal tubercle. **d**: Schematic diagram of the location of the U-shaped internal fixation. The internal fixation was located in the soft tissue space anterior to the Achilles tendon

A 4–5 cm U-shaped incision was made on the horizontal plane at the level of the calcaneal tubercle after general anesthesia. Gradually separate the soft tissue according to the anatomical structure, and expose the calcaneal tubercle fracture on the premise of protecting the Achilles tendon, peripheral blood vessels and other tissues as much as possible. Then we reduced the fracture block under the naked eye and temporarily fixed it with Kirschner wires. The next is the most critical step. By making a soft tissue channel in front of the Achilles tendon stop, the pre-curved micro locking plate (Weigao, Shandong, China) was U-shaped fixed on both sides of the calcaneal tubercle with the locking screws. If the fracture block was large and the traction force of Achilles tendon was strong, we can add another micro locking plate to increase stability and firmness (Fig. 1).

### Postoperative management

The ankle brace was need for three weeks after surgery. The patient began to ankle exercise from the first day after surgery by guiding of the surgeon. After 3 weeks, the patients can do some daily exercises and walk with weight under the protection of crutches. Patients were encouraged to walk with full weight bearing at 6 weeks postoperatively and to participate in normal daily physical exercise at 12 weeks postoperatively. After 12 months, the internal fixation was always removed.

### Clinical evaluation

The regular follow up times were 1, 3 ,6 and 12 months. The maintenance of the plate and the fracture were evaluated by the routine anteroposterior and lateral imaging of the heel. The American Orthopaedic Foot and Ankle Association (AOFAS) score, Cedell score and the visual analog scale (VAS) were used to evaluate functional results [19–21].

### Statistics

Mean ± standard deviation (SD) was used to express the numerical data. AOFAS score before and after surgery was compared by Paired t test. The software SPSS 20.0 was used for statistical analysis, and a P value of less than 0.05 was considered significant.

## Results

### General results

In this study, 9 male and 7 female with the average age of 52.9 years were included. About the injury-to-surgery interval (ISI), there were 3.3 days. The operation time were 55.1 min. All patients were followed up for an average of 12.6 months (Table 1).

**Table 1:** Patient Information

	Gender	Age (years)	Fracture type	Follow up time (months)
Case 1	male	53	Beavis I I	12
Case 2	female	68	Beavis I	12
Case 3	female	54	Beavis I	11
Case 4	male	46	Beavis I I	12
Case 5	male	43	Beavis I I	13
Case 6	male	59	Beavis I I	12
Case 7	female	38	Beavis I I	15
Case 8	female	66	Beavis I	11
Case 9	male	42	Beavis I I	15
Case 10	male	39	Beavis I I	13
Case 11	female	49	Beavis I I	12
Case 12	male	57	Beavis I	13
Case 13	female	48	Beavis I I	13
Case 14	male	56	Beavis I I	10
Case 15	male	65	Beavis I	13
Case 16	female	63	Beavis I	15

### Functional results

**Fig. 2:**
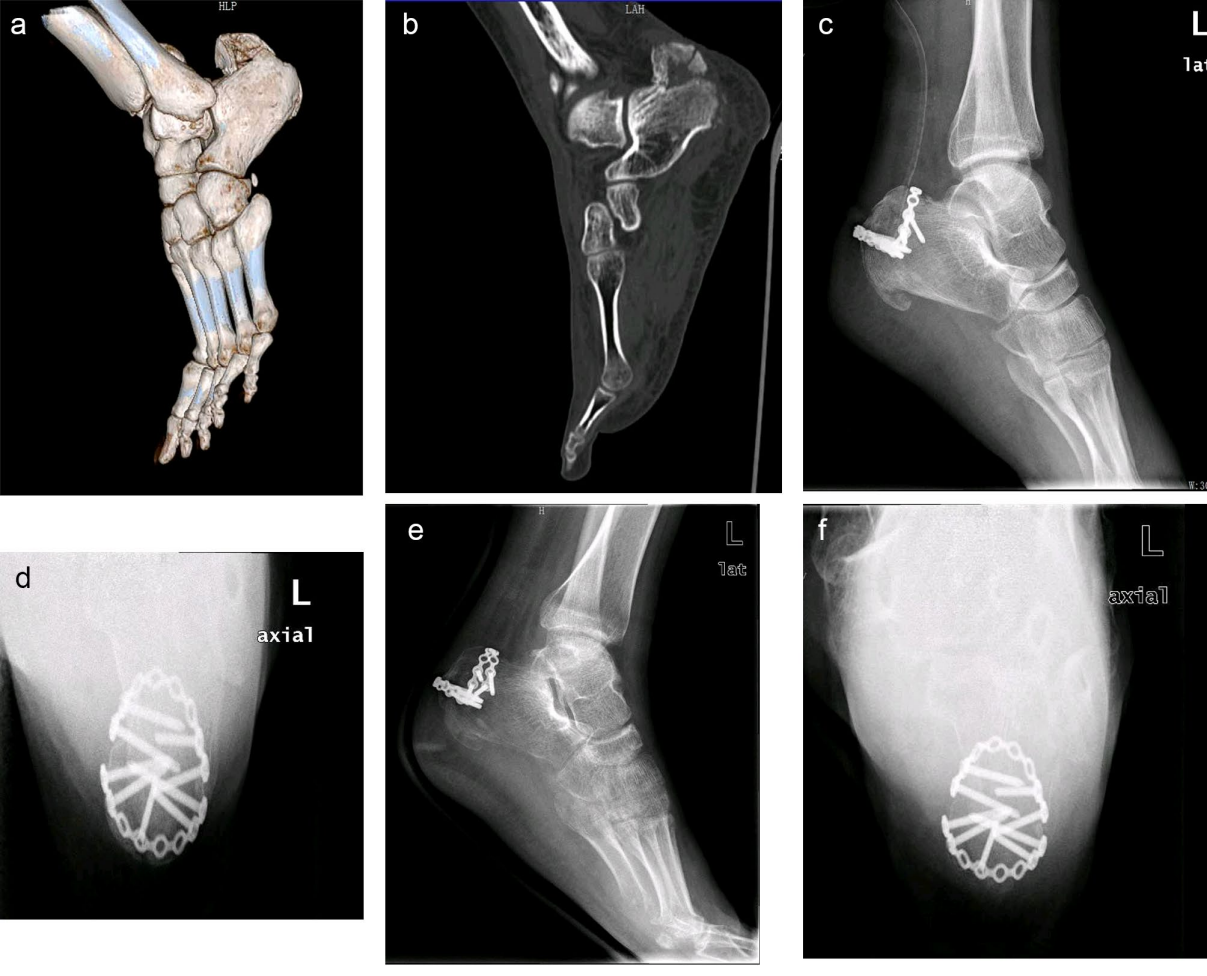
**a.b**: CT scan of the calcaneus plus three-dimensional reconstruction, the fracture of calcaneal tuberosity with obvious displacement. **c.d**: Lateral and axial films of calcaneus 3 days after operation. Two micro locking plates were used to fix the fracture block from the back of Achilles tendon insertion point and in front of Achilles tendon insertion point respectively. The reduction of fracture block was good and the internal fixation was firm. **e.f**: Lateral and axial films of calcaneus half a year after operation. The fracture healed well without complications

The preoperative AOFAS score was 26.34 ± 3.34, which was significantly different from 91.38 ± 6.15 half a year after operation (p = 0.003). The preoperative Cedell score was 31.05 ± 4.18 and the score half a year after operation was 92.17 ± 5.39. It was also considered that there was a significant difference in statistical analysis (p = 0.011). The VAS score was 8.91 ± 1.51 before operation and decreased to 0.58 ± 1.31 half a year after operation (p = 0.014) (Table 2). All patients got bone healing. There were no postoperative infection, hardware failure and fractures of calcaneal tubercle (Fig. 2).

**Table 2:** Functional results

	Before surgery	Six months after surgery	P-value
AOFAS Score	26.34 ± 3.34	91.38 ± 6.15	p = 0.003
CEDELL Score	31.05 ± 4.18	92.17 ± 5.39	p = 0.011
VAS Score	8.91 ± 1.51	0.58 ± 1.31	p = 0.014

## Discussion

The calcaneal tubercle is the attachment point of the Achilles tendon, and its upper edge forms an angle of 30 ~ 45 degree with the talocalcaneal articular surface (Bohler angle). When the displacement of calcaneal tubercle fracture is greater than 6 mm, the angle will shrink or disappear, and even become a negative angle in severe cases. This will change the lever of the Achilles tendon and cause significant changes in the load and contact point of the subtalar joint. It is easy to develop into large painful protrusions and cause persistent pain and early traumatic arthritis [22]. Therefore, open reduction and internal fixation is the first choice in the treatment scheme. The key to treatment is to restore the counterpoint relation of subtalar joint and Bohler angle.

The surgical treatment of calcaneal tubercle fracture includes screw fixation, plate fixation, Kirschner wire fixation, anchor fixation, external fixation and fracture fragment resection [23–25]. Giordano et al. [26] reported that calcaneal tuberosity fractures were more common in osteoporotic patients, such as the elderly, diabetes and autoimmune diseases. Osteoporosis makes fixation more difficult. Some researchers advocated that the fracture should be treated by lag screw fixation or combined with anchor and wire fixation through the distal lateral or medial approach of Achilles tendon [27]. Although lag screw fixation is the most widely accepted surgical method for the treatment of this fracture, we found a high rate of re-displacement of fracture in early follow-up. At the same time, the fracture site needed to be fixed for a long time, which was easy to lead to the loss of ankle dorsiflexion angle and the decline of heel lifting function of Achilles tendon in the later stage [28]. The hoop wire based on the principle of tension band fixation effectively neutralizes the upward traction of Achilles tendon. It is a simple and easy fixation method, which has advantages compared with other fixation methods. However, some scholars also reported that the loosening and stimulation of internal fixation of posterior calcaneal tubercle caused skin complications and discomfort as high as 64% in the later follow-up [29]. The anchor combined with lag screw strengthens the lag screw fixation of fracture and neutralizes the tension of Achilles tendon. Compared with single screw fixation, the load of this reinforcement method is almost twice that of screw fixation. However, this method needs to pull the tendinous part of the Achilles tendon, which is prone to complications such as Achilles tendon contracture and loss of heel lifting function of Achilles tendon in varying degrees in the later follow-up [30]. On the one hand, calcaneal tubercle is vulnerable to strong traction of Achilles tendon. On the other hand, calcaneus is cancellous bone and the strength of screw fixation is limited, which is prone to re-displacement of fracture and fixation failure. How to avoid local skin necrosis, internal fixation failure, local irritation discomfort at Achilles tendon stop point and loss of heel lifting function of Achilles tendon? How can we carry out early functional exercise after treatment? These are difficult points in the clinical treatment of calcaneal tubercle fracture.

The treatment method of U-shaped fixation (180-degree fixation) of calcaneal tubercle fracture with micro locking plate was first proposed by us in early 2021, which provides a new treatment scheme for clinicians. After early case collection and data analysis, we found that its clinical efficacy was excellent, and there were no obvious complications after operation. It was a safe treatment for calcaneal tubercle fracture. Compared with traditional surgery, this method adopted U-shaped incision which was different from traditional straight and L-shaped incision. Some researchers believed that the soft tissue covering Achilles tendon and calcaneal tubercle was thin and the blood supply was unstable. If the operation at this site encountered some complications of the wound, it was a devastating problem for the operator. Using U-shaped incision at the level of calcaneal tubercle, the surgical scars were more aesthetically pleasing, less prone to scars contracture, and conducive to faster wound healing process. These might be due to the fact that the incision was parallel to the skin lines. In addition, the incision could directly expose the avulsed bone fragments, which was conducive to the operation [31]. Our study also found that the incision had little invasion to the soft tissue, and the incision along the transverse lines of the skin was more conducive to the beauty of the postoperative scar. At the same time, the postoperative scar had no contracture and little effect on the ankle movement. The volume of the micro steel plate used in our operation was small, and the probability of compression on the heel skin was almost zero. During our follow-up for more than one year, no similar compression was found. There was a certain space between the Achilles tendon stop and the posterior calcaneal tubercle, which was enough to meet the placement of 1 to 2 micro plates. If the fixation strength of one steel plate was not enough, a micro steel plate (Fig. 2) could be added behind the Achilles tendon stop for the cases with large fracture block and obvious displacement, and there was no need to worry about the incision caused by its compression on the skin. Calcaneus was composed of cancellous bone, which was not an ideal fixation site and was easy to cause loosening and failure of internal fixation [32]. Although the calcaneal tubercle was the site with good bone quality and mechanical properties in the whole calcaneus [33], the probability of internal fixation failure was not low due to the strong traction of Achilles tendon [14, 15]. We need a mechanically stronger fixation method in clinic. U-shaped locking steel plate fixation meets this demand to a certain extent. Relying on the good plasticity of the micro locking plate, we can pre bend and shape the plate according to the specific shape of the fracture block during the operation. We need to place the plate as close to the cortical bone of the calcaneal tubercle as possible, and bend it to form a “U” like structure to wrap the fracture block of the posterior calcaneal tubercle. The U-shaped (180 degree) cross screw placement method is used for bicortical locking fixation with sufficient screws to provide more powerful mechanical strength, which can greatly strengthen the firmness and reliability of fixation and achieve the purpose of early postoperative functional exercise. It is especially suitable for elderly patients with osteoporosis. The results of our study also confirmed the reliability of this method. None of the 16 patients underwent revision surgery because of internal fixation failure. The excellent efficacy was also illustrated by the functional scores at the postoperative follow-up. The AOFAS score was 91.38 ± 6.15 half a year after operation. Significant improvement in postoperative AOFAS functional scores compared to research treated the calcaneal tubercle fracture with screws and plates [14, 34]. Beavis III fracture was not included in this study. The main consideration is that the operation method we recommend is mainly suitable for fracture types with a certain volume of fracture blocks. Micro plates and locking screws can be well fixed. For Beavis III fractures, anchors may be a more appropriate fixation method [35].

### Strengths and limitations

U-shaped fixation is a new treatment for calcaneal tubercle fracture. Through more than 2 years of case collection, we found that the therapeutic effect is positive and is a recommended treatment. However, the number of cases in our study needs to be increased, and there is a lack of prospective comparative study. In addition, the biomechanical research of U-shaped fixation is still blank, and we need basic mechanical research to verify the mechanical advantages of this fixation scheme.

## Conclusions

In the treatments of calcaneal tubercle fracture, U-shaped internal fixation is a new attempt. Through the short-term follow-up study, we found that its therapeutic effect is excellent, which is a recommended treatment in clinic.

## Data Availability

The datasets used and/or analyzed during the current study are available from the corresponding author on reasonable request.

## Abbreviations

AOFAS: American Orthopaedic Foot and Ankle Association score
VAS: Visual analog scale
ISI: Injury-to-surgery interval
